# 496. Maternal Participation in Enrollment Interview and Risk of Bias in a Multicenter Pediatric Network Evaluating COVID-19 Vaccine Effectiveness in Infants

**DOI:** 10.1093/ofid/ofad500.565

**Published:** 2023-11-27

**Authors:** Regina Simeone, Laura D Zambrano, Margaret M Newhams, Natasha B Halasa, Katherine E Fleming-Dutra, Amber Orzel, Michael J Wu, Adrienne G Randolph, Angela P Campbell

**Affiliations:** Centers for Disease Control and Prevention, Atlanta, Georgia; Centers for Disease Control and Prevention, Atlanta, Georgia; Boston Children's Hospital, Boston, Massachusetts; Vanderbilt University Medical Center, Nashville, Tennessee; Centers for Disease Control and Prevention, Atlanta, Georgia; Boston Children's Hospital, Boston, Massachusetts; Centers for Disease Control and Prevention, Atlanta, Georgia; Boston Children's Hospital, Harvard Medical School, Boston, Massachusetts; Centers for Disease Control and Prevention, Atlanta, Georgia

## Abstract

**Background:**

The Overcoming COVID-19 network uses a case-control design to estimate effectiveness of maternal COVID-19 vaccination on COVID-19 associated hospitalization of infants. Vaccination history is asked in a maternal interview and verified with jurisdictional immunization information systems (IIS). In the absence of an interview, IIS data alone are used. These data may become less complete as the public health emergency ends and COVID-19 vaccines move to the commercial market. Vaccination status may rely more on interviews, potentially biasing VE estimates. We analyzed possible future participation bias.

**Methods:**

Maternal interview participation was assessed for 699 case infants < 6 months hospitalized with COVID-19 and 613 SARS-CoV-2 test-negative control infants < 6 months with COVID-like illness admitted to 29 U.S. hospitals from December 19, 2021–January 31, 2023. To assess recent changes in participation, two Omicron subvariant periods were defined: Period 1 (December 19, 2021–July 18, 2022) and Period 2 (July 19, 2022–January 31, 2023). Interview participation percentages for vaccinated and unvaccinated cases and controls, overall and by subvariant, were used to calculate interview participation bias factors (BF) (Table 1). The BF can be used to adjust observed estimates of VE for potential participation bias.

**Figure:**
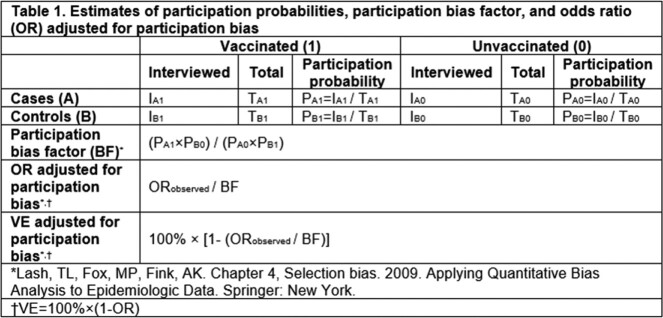

**Results:**

Overall, vaccination was more frequent among participating case (25%) vs. non-participating case (16%) mothers (Table 2). Vaccination status did not differ by interview participation among controls. Participation was highest in Period 1 (68% in vaccinated cases) and lowest in Period 2 (31% in vaccinated controls) (Table 3). During Period 1, the BF was 1.0, suggesting no participation bias would occur by limiting to interviewed participants. During Period 2, the BF was 1.9. Independent of other possible biases, differences in participation by case/control and vaccination status would be biased by a factor of 1.9, lowering the observed VE.

**Figure:**
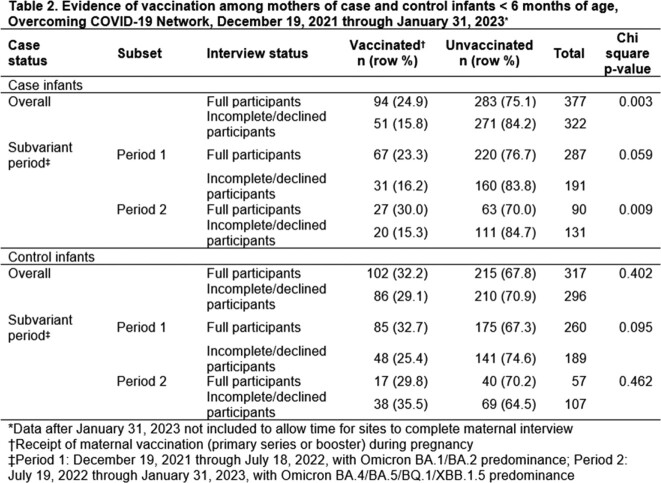

**Figure:**
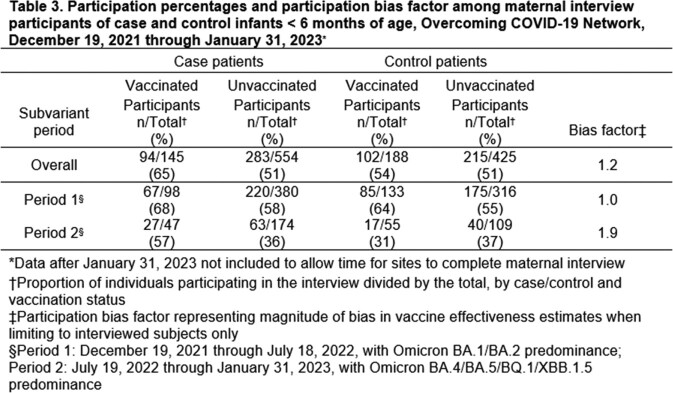

**Conclusion:**

Participation in the interview differed by case/control and vaccination status during recent Omicron subvariant circulation. Independent of other biases, our results suggest that future estimates of maternal VE could be biased if future estimates of maternal VE include only women who complete an interview.

**Disclosures:**

**Regina Simeone, PhD**, Pfizer: Stocks/Bonds **Natasha B. Halasa, MD, MPH**, Merck: Grant/Research Support|Quidell: Grant/Research Support|Quidell: donation of kits|Sanofi: Grant/Research Support|Sanofi: vaccine support

